# Congenital adrenal hyperplasia is a very rare cause of adrenal incidentalomas in Sweden

**DOI:** 10.3389/fendo.2022.1017303

**Published:** 2022-12-05

**Authors:** Fredrik Sahlander, Sophie Bensing, Henrik Falhammar

**Affiliations:** ^1^ Department of Molecular Medicine and Surgery, Karolinska Institutet, Stockholm, Sweden; ^2^ Department of Medicine, Falu Hospital, Falun, Sweden; ^3^ Center for Clinical Research Dalarna-Uppsala University, Falun, Sweden; ^4^ Department of Endocrinology, Karolinska University Hospital, Stockholm, Sweden

**Keywords:** adrenal tumor, 21-hydroxylase deficiency, 17-hydroxyprogesterone, adrenocorticotropic hormone, *CYP21A2* variations

## Abstract

**Background:**

Undiagnosed congenital adrenal hyperplasia (CAH) can cause adrenal incidentalomas, but the frequency is unclear.

**Objectives:**

This study aimed to investigate the prevalence of CAH in a population with adrenal incidentalomas and report the clinical characterization.

**Material and methods:**

This was a prospective study performed at a regional hospital from 2016 to 2021. Patients with adrenal incidentalomas were investigated with an adrenocorticotropic hormone (ACTH)-stimulation test in addition to hormonal workup. Serum cortisol and 17-hydroxyprogesterone (17OHP) were analyzed. Individuals with a basal or stimulated 17OHP ≥30 nmol/L were classified as suspicious non-classic CAH, and a *CYP21A2*-gene analysis was performed in these subjects.

**Results:**

In total, 320 individuals with adrenal incidentalomas were referred to the center, and of these individuals, an ACTH-stimulation test was performed in 222 (median age, 67 (24–87) years; 58.6% women; and 11.7% with bilateral lesions). None of the individuals presented a basal 17OHP ≥30 nmol/L, but there were 8 (3.6%) who did after ACTH stimulation. Four of these subjects (50%) presented bilateral lesions, and the tumor size was larger compared to that of the individuals with a stimulated 17OHP <30 nmol/L (median, 38 (19–66) vs. 19 (11–85) mm, p=0.001). A *CYP21A2* variation (p.Val282Leu) was detected in one of the eight subjects with a stimulated 17OHP ≥30 nmol/L, i.e., the patient was a heterozygotic carrier. None of the eight subjects presented with cortisol insufficiency or clinical signs of hyperandrogenism.

**Conclusions:**

The prevalence of non-classic CAH in an adrenal incidentaloma cohort was 3.6% based on stimulated 17OHP and 0% based on gene analysis. CAH should be considered in AI management in selected cases and confirmed by genetic analysis.

## Introduction

Adrenal masses are very common accidental findings during imaging procedures and are reported in approximately 1%–5% of computer tomography (CT) and magnetic resonance imaging (MRI) scans visualizing the adrenal glands (1). The term adrenal incidentaloma (AI) was coined by Geelhoed and Druy as early as 1982 (2). AI is now established as one of the most common endocrine diagnoses, and the clinical management of AIs is probably one of the most expanding fields in endocrinology during the last decades (3, 4). A development with improved imaging technology, awareness of the diagnosis among radiologists, and availability of CT and MRI scanners in healthcare service are factors contributing to the increased number of cases diagnosed with AIs (4–6).

AIs may be a threat to health if they are malignant or exhibiting autonomous hormone production. Adrenocortical carcinomas (ACCs) are reported in 1.2%–11% of AIs and metastases of extra-adrenal origin in up to 20% in patients with a history of extra-adrenal malignancies (7, 8). Approximately 10%–15% of investigated AIs present hormone excess (1). A number of studies have shown an association of AIs with increased morbidity and mortality (9–13), mainly due to hormone excess. Therefore, the clinical management of AIs aims to rule out malignancy and hormone overproduction, which can pose a health risk over time. The hormonal workup recommended by the European Society of Endocrinology (ESE) and the European Network for the Study of adrenal tumors (ENSAT) is quite straight forward in the majority of cases (8), but there are still areas with unclear management in the setting of AIs. One such area is congenital adrenal hyperplasia (CAH), which is a condition associated with AIs (14).

CAH is a group of recessive inherited enzyme defects in the synthesis of glucocorticoids and mineralocorticoids in the adrenal cortex (15). Different enzymes are affected, leading to different clinical phenotypes with different degrees of glucocorticoid and mineralocorticoid insufficiency and hyper- or, in some rare forms, hypoandrogenism (15–17). 21-Hydroxlase is the most common affected enzyme in CAH, which is reported in up to 99% of all cases (15, 18). Different *CYP21A2*-gene variations cause varying degrees of 21-hydroxylase deficiency (21OHD) (19). CAH is usually divided into three clinical phenotypes. Salt-wasting (SW) CAH is the most severe form with combined life-threatening glucocorticoid and mineralocorticoid insufficiency and elevated androgens, while simple virilizing (SV) is associated with a milder mineralocorticoid insufficiency but with a pronounced glucocorticoid insufficiency and elevated levels of androgens (20). In contrast, the mildest form, non-classic (NC) CAH, presents with a mild, often subclinical hypocortisolism and mildly elevated androgens (21). The two more severe phenotypes, i.e., classic CAH, are nowadays in many countries diagnosed in the neonatal period by neonatal screening (20, 22). The diagnosis of NC CAH is often missed in the neonatal screening program and normally present, if ever, in adolescence or young adulthood (21). The true prevalence of NC CAH is unknown but is estimated to be approximately 0.1% in the general population and up to 2%–4% in some ethnical populations (23). The CAH diagnosis is based on biochemical findings; an elevated basal serum 17-hydroxyprogesterone (17OHP) is obligate in untreated classic forms of CAH, while in NC CAH, basal serum 17OHP is sometimes not elevated, so to exclude or confirm NC CAH stimulated 17OHP [with an adrenocorticotropic hormone (ACTH)-stimulation test] is required (15, 20, 23). The diagnosis could then be confirmed by *CYP21A2*-gene variation analysis (21).

Adrenal tumors are common in individuals diagnosed with CAH, with reported frequencies approximately 30% (24). The hypothesis is that chronic elevated levels of ACTH have a trophic and proliferative effect on the adrenal cortex, leading to development of cortical hyperplasia and tumor development (14). This hypothesis is supported by reports finding that adrenal tumors are more common in non-compliant patients, where deficient glucocorticoid intake leads to higher ACTH levels (24). Myelolipomas is a form of adrenal tumor clearly associated with CAH (25), and approximately 25% of the adrenal lesions in CAH were reported as myelolipomas (24).

The ESE/ENSAT guidelines recommend CAH to be considered in case of bilateral lesions (8). There are still knowledge gaps regarding the relationship between CAH and adrenal lesions that needs to be clarified (24). The prevalence of CAH in an unbiased population with AI is not known, and there is a need to further characterize clinical, radiological, and biochemical features of AIs related to CAH (26).

The objective of this study was to investigate the prevalence of CAH in a population with adrenal incidentaloma using the ACTH-stimulation test as screening method and then genetic confirmation. Secondary aims were clinical, radiological, and biochemical characterizations of subjects with CAH.

## Material and methods

Individuals 18 years or older who were diagnosed with adrenal masses during the inclusion period (2016–2021), fulfilling the European Society of Endocrinology’s definition of adrenal incidentaloma (8), were consecutively included at Falu Hospital, a regional hospital in Sweden. The study was approved by the Regional Ethical Review Board in Uppsala, Sweden [diary number 2015/096 (2019-04522)], and written informed consent was obtained from all subjects.

All subjects were radiological and hormonally investigated in accordance with the European Society of Endocrinology’s 2016 guidelines for clinical management of adrenal incidentalomas (8).

The radiological investigations, CT scans, were performed at any of the region’s four local hospitals. To reduce the risk of including subjects not meeting the criteria of AI, all images and imaging reports were reviewed. Only adrenal lesions ≥10 mm were included.

All subjects were investigated at the regional hospital (Falu Hospital in the Region of Dalarna in Sweden) performing physical status (weight, length, and blood pressure) and blood sampling. The subjects fasted from midnight the day before the visit. All blood samples were collected from 8 to10 a.m. Blood samples were obtained for the analysis of plasma renin, plasma aldosterone, and plasma metanephrines. In addition to the routine hormonal workup, serum dehydroepiandrosterone sulfate (DHEAS), serum cortisol, and serum 17OHP levels were analyzed.

An ACTH-stimulation test was then performed by the administration of 0.25 mg tetracosactide (Synacthen^®^) intravenously. Blood samples for analysis of serum cortisol and 17OHP were extracted at baseline and 30 and 60 min after the injection. The maximum values of serum cortisol and 17OHP after ACTH stimulation were used in the data analysis, regardless of whether the highest value was reached at 30 or 60 min after administration of tetracosactide.

A basal or stimulated serum 17OHP ≥30 nmol/L was classified as NC CAH in accordance with current guidelines (20). The population was stratified in two groups, one with a serum 17OHP ≥30 nmol/L (suspected NC CAH) and the other with serum 17OHP <30 nmol/L, for the comparison of radiological, clinical, and biochemical characteristics. A *CYP21A2*-gene analysis was performed in subjects with serum 17OHP ≥30 nmol/L to confirm the CAH diagnosis.

Finally, an overnight dexamethasone suppression test (DST) was performed in all subjects with a margin of at least 14 days after the visit in order not to risk the DST being affected by the ACTH-stimulation test. One milligram of dexamethasone was administrated per oral at 11 p.m., and a blood sample was extracted at 8 a.m. the day after to analyze serum cortisol. Late-night salivary cortisol (LNSC) was performed at 11 p.m., and diurnal urine-free cortisol (UFC) was performed in subjects with serum cortisol >50 nmol/L after DST. In subjects with serum cortisol >138 nmol/L, a plasma ACTH and serum cortisol were performed at 8 a.m. Subjects classified as having autonomous cortisol secretion (ACS), in accordance with ESE/ENSAT guidelines (serum cortisol >138 nmol/L after overnight 1 mg dexamethasone) with elevated LNSC and/or diurnal UFC, were referred to a multidisciplinary therapy conference (MDTC) to assess indication of adrenalectomy.

### Hormonal assays

Serum cortisol was analyzed with competitive chemiluminescence immunoassay (instrument Siemens ADVIA Centaur XP and Siemens Atellica IM 1600; reagent kit Cortisol (COR) product number 10629846 and 10995336, respectively). Serum 17OHP levels were analyzed with competitive ELISA (instrument Wallac viktor 2 (Perkin Elmer); reagent kit IBL International) and ultra-high performance liquid chromatography coupled with tandem mass spectrometry (UHPLC-MS/MS) (instrument Waters Xevo TQ MS, Waters Xevo TQ-S and Waters Xevo TQ-S micro; no reagent kit).

Plasma renin was analyzed by non-competitive immunometry with chemiluminescence detection (instrument Liaison XL; reagent kit Diasorin), plasma aldosterone with competitive immunometry with chemiluminescence detection (instrument Liaison XL; reagent kit Diasorin), serum DHEAS with competitive electrochemiluminescence immunoanalysis (ECLIA) (instrument Roche cobas e602 and Roche cobas e801; reagent kit Roche Elecsys DHEA-S), and plasma metanephrines using an LC-MS/MS-system using a HILIC column and a triple quadrupole mass spectrometer (6495B, Agilent) after purification by a reversed-phase solid-phase extraction (Phenomenex) with the addition of deuterium (d_3_)-labeled internal standards (Medical Isotopes/Larodan).

Complementary analyses in subjects classified as possible ACS (PACS) and ACS were performed with UFC (high-resolution mass spectrometry (instrument Thermo Fischer Orbitrap Q-Exactive; reagent kit in-house)), LNSC (competitive electrochemiluminiscence immunoanalysis (ECLIA) (instrument Roche cobas e602 and Roche cobas e801; reagent kit Roche Elecsys Cortisol II)), serum cortisol (see above), and plasma ACTH (non-competitive immunometry with chemiluminescence detection (instrument Immunlite 2000 XPi; reagent kit Diasorin). DNA was extracted from EDTA-anticoagulated whole blood in subjects with 17OHP levels ≥30 nmol/L. Coding regions and splice sites of *CYP21A2* were sequenced in clinical laboratories using either targeted next-generation sequencing (NGS) or Sanger sequencing. Deletions and duplications were sought using multiplex-ligation-dependent probe amplification (MLPA) or NGS-based del/dup analysis of *CYP21A2*. Mutations were classified according to ACMG guidelines and reported with HGVS nomenclature (www.hgvs.org). A molecular genetic diagnosis of CAH due to 21OHD was reserved for cases with biallelic loss-of-function mutations.

### Statistical analysis

Statistical analysis of the data was performed using IBM SPSS Statistics for Windows, Version 28.0.0.0 (190), released 2021, IBM Corp., Armonk, NY. Continuous data with a normal distribution and homogeneity of variance are presented as the mean ± standard deviation. An unpaired t-test was used for comparisons between the two groups if normally distributed and Mann–Whitney U test if not normally distributed. Continuous data without normal distribution and homogeneity of variance are presented as median (range). Non-parametric tests were then used for comparisons between the groups. Categorical variables are presented as counts and percentages, and comparisons between the two groups were performed using the Fisher’s exact test. The Pearson test was used to analyze correlations between continuous variables. Two-sided p-values <0.05 were considered statistically significant.

## Results

In total, 320 individuals were referred to the center for investigation of adrenal incidentalomas, of which 228 subjects were assessed to meet the criteria for inclusion and underwent blood sampling and ACTH-stimulation test. Six subjects were excluded after review of the images, as the lesions did not meet the criteria for AI. In two of the subjects, the lesions were too small after repeated size assessment, and in two, the lesions were of extra-adrenal localization after review. In the final two excluded subjects, the lesions turned out to be adrenal hemorrhages and went into complete regression after repeated imaging. None of the excluded subjects presented a basal or stimulated 17OHP ≥30 nmol/L or any biochemical signs of hormonal excess.

After exclusion, 222 subjects remained. The median age was 67 (24–87) years, 58.6% were women, and 94.1% were of Swedish ethnicity. None of the subjects presented a basal 17OHP <30 nmol/L.

The population was stratified in two subgroups based on level of ACTH-stimulated serum 17OHP, <30 and ≥30 nmol/L, respectively. Eight subjects presented a stimulated 17OHP level ≥30 nmol/L. In **Table 1**, radiological, clinical, and biochemical characteristics of the eight subjects with a serum 17OHP level ≥30 nmol/L are presented.

**Table 1:** Characteristics of the eight subjects with an adrenocorticotrophic hormone (ACTH)-stimulated serum 17-hydroxyprogesterone (17OHP) level ≥30 nmol/L.

Subject (number)	Age (years)	Gender (male/female)	Ethnicity	Tumor size (mm)	Tumor localization (uni-/bilateral)	Attenuation ( HU)	Basal serum cortisol (nmol/L)	Max stimulated serum cortisol (nmol/L)	Basal serum 17OHP (nmol/L)	Max stimulated serum 17OHP (nmol/L)	Pathological *CYP21A2*-variations	Serum DHEAS (nmol/L)
1	44	female	Swedish	45	unilateral	<10	320	750	3.3	50	No	0.42 (1.6-9.2)
2	56	female	Swedish	19	unilateral	<10	315.6	1130.4	5.6	35	No	0.54 (0.5-5.6)
3	60	female	Ukrainian	30	unilateral	<10	594.6	1259	1.9	33	No	2.2 (0.5-5.6)
4	62	male	Swedish	20	unilateral	<10	410	1040	3.4	34	No	1.21 (1.4-8.0)
5	66	female	Swedish	56	bilateral	<10	598.3	2205.1	1.6	39	No	3.5 (0.3-6.7)
6	66	female	Scandinavian	66	bilateral	<10	356.9	1498.2	0.9	62	Heterozygote c.844G>T (p.Val282Leu)	0.99 (0.3-6.7)
7	70	male	Swedish	41	bilateral	<10	386.9	1307.9	2.4	37	No	1.19 (0.9-6.8)
8	79	male	Swedish	35	bilateral	<10	510	960	5	49	No	0.57 (0.4-4.3)

There was no difference between the levels of basal serum cortisol between the two groups (400.0 (97.7–930.0) vs. 398.5 (315.6–598.3) nmol/L, p=0.429). Basal and stimulated serum 17OHP levels and stimulated serum cortisol levels were all significantly higher in the subgroup with 17OHP levels ≥30 nmol/L. Comparing the stimulated 17OHP levels <30 group vs. 17OHP levels ≥30 nmol/L group showed basal 17OHP levels of 1.1 (0.3–5.5) vs. 2.9 (0.9–5.6) nmol/L (p =0.003), stimulated serum 17OHP levels of 8.9 (2.4–27.0) vs. 38.0 (33.0–62.0) nmol/L (p <0.001), and stimulated serum cortisol levels of 980.0 (347.1–2,000.0) vs. 1,194.7 (750.0–2,205.1) nmol/L (p=0.030). One subject with serum 17OHP levels <30 nmol/L after ACTH stimulation had a partial cortisol insufficiency (maximum serum cortisol level, 347 nmol/L). The partial insufficiency was explained by a prolonged treatment with per oral glucocorticoid (prednisolone) due to an inflammatory bowel disease. This subject presented a maximum serum 17OHP level after ACTH stimulation of 7.7 nmol/L.

There was a difference in tumor size when comparing the groups with those with high 17OHP having a maximum diameter of 38 (19–66) mm compared with 19 (11–85) mm in the others (p=0.001). The frequency of bilateral lesions was also higher in the elevated 17OHP group (50% vs. 10.3%, p=0.008). In **Table 2**, a summary of clinical, radiological, and biochemical data for the whole population and the two groups is presented.

**Table 2:** Characteristics of the whole population and the two subgroups (adrenocorticotrophic hormone [ACTH]-stimulated serum 17-hydroxyprogesterone [17OHP] levels ≥30 nmol/L and <30 nmol/L respectively).

	All subjects (n=222)	Serum 17OHP ≥30 nmol/L (n=8)	Serum 17OHP <30 nmol/L (n=214)	P-value
Age (median (min-max))	67 (24-87)	64 (44-79)	67 (24-87)	0.543
Female gender (%)	58.6	62.5	58.4	0.561
Swedish ethnicity (%)	94.1	75	94.9	0.073
Tumor size (mm, median (min-max))	19.5 (11-85)	38 (19-66)	19 (11-85)	0.001
Bilateral lesions(%)	11.7	50	10.3	0.008
Basal serum cortisol (nmol/L (median (min-max))	400.0 (97.7-930.0)	398.5 (315.6-598.3)	400.0 (97.7-930.0)	0.429
Max stimulated serum cortisol (nmol/L (median (min-max))	991.8 (347.1-2205.1)	1194.7 (750.0-2205.1)	980.0 (347.1-2000.0)	0.030
Basal serum 17OHP (nmol/L (median (min-max))	1.1 (0.3-5.6)	2.9 (0.9-5.6)	1.1 (0.3-5.5)	0.003
Max stimulated serum 17OHP (nmol/L (median (min-max))	9.2 (2.4-62.0)	38.0 (33.0-62.0)	8.9 (2.4-27.0)	<0.001

P-value for comparison of the two subgroups.

In the eight subjects with a serum 17OHP level ≥30 nmol/L, an analysis of the *CYP21A2* gene was performed. In seven of eight subjects (87.5%), no pathological *CYP21A2* variations were detected.

In one subject with an ACTH-stimulated serum 17OHP level ≥30 nmol/l, a heterozygotic pathological *CYP21A2* variation, c.844G>T (p.Val282Leu), was detected. This subject exhibited a stimulated serum cortisol level of 1,498 nmol/L and a stimulated serum 17OHP level of 62 nmol/L and had bilateral adrenal lesions (maximum diameters, 49 and 17 mm) with an attenuation of <10 Hounsfield units (HU). Biochemically, this subject was classified as ACS after DST (serum cortisol, 284.8 nmol/L). Further investigation of mild adrenal cortisol excess with repeated normal daily urinary-free cortisol level showed that it did not suppress plasma ACTH level (1.5pmol/l (<10)) or serum DHEAS level (0.99 umol/l (0.3–6.7)). Salivary cortisol levels showed a flat diurnal cortisol rhythm. Unilateral adrenalectomy was discussed at a multidisciplinary therapy conference but was rejected due to pronounced overweight of the subject.

**Figure 1:**
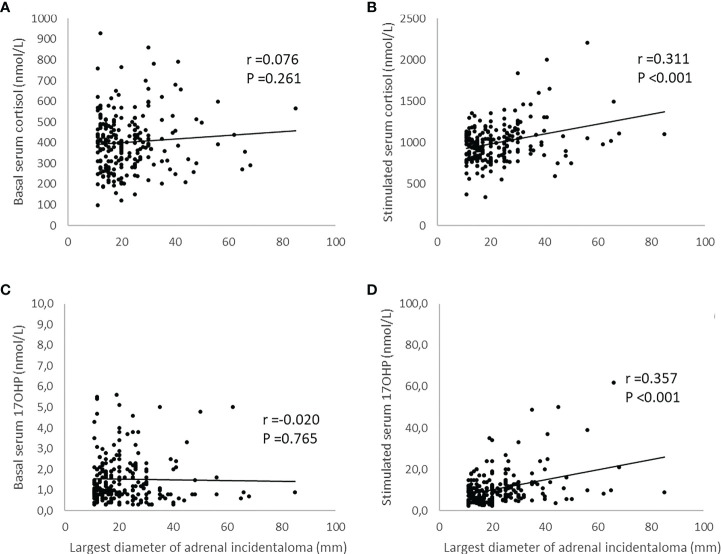
Illustration of the relationship between largest diameter of adrenal incidentaloma and levels of serum cortisol and serum 17-hydroxyprogesterone (17OHP) before and after adrenocorticotrophic hormone (ACTH)-stimulation. The simulated levels of serum cortisol and serum 17OHP were obtained 30 and 60 minutes after administration of 0.25 mg tetracosactide (Synacthen® 521 ) intravenously. The maximum value was then used to investigate the relationship using Pearson´s test. Panels **(A, C)** show not significant correlations between tumor size and basal serum cortisol and tumor size and basal serum 17OHP respectively. Panel **(B)** shows a significant correlation between tumor size and stimulated serum cortisol and panel **(D)** a significant correlation between tumor size and stimulated serum 17OHP.

As no cases with pathological *CYP21A2* variations in both alleles could be identified, correlations between tumor size and basal levels of serum 17OHP and serum cortisol were examined in the entire population and not the two subgroups. There were no significant correlations between tumor size and basal cortisol levels (R=0.076, p=0.261) and tumor size and basal serum 17OHP levels (R=−0.020, p=0.765). The correlations between tumor size and stimulated serum cortisol levels and tumor size and stimulated serum 17OHP levels were significant (R=0.311, p <0.001 and R=0.357, p <0.001, respectively) (**Figure 1**).

A serum DHEAS level in the normal range was observed in 82.8% (183 of 221) of the subjects, a value below normal range in 14.9% (33 of 221), and an elevated DHEAS level in 2.3% (5 of 221). Of the eight subjects with 17OHP levels ≥30 nmol/L, no subject presented an elevated DHEAS, 25% (2 of 8) a subnormal, and 75% a DHEAS level in the normal range (6 of 8). There was no significant correlation between DHEAS levels and stimulated 17OHP levels (R=−0.131, p=0.052) and DHEAS and stimulated cortisol levels (R=−0.124, p=0.066).

## Discussion

The main goal of this study was to investigate the prevalence of CAH using the ACTH-stimulation test as a screening method and gene analysis to confirm the diagnosis. A prevalence of 3.4% using the ACTH-stimulation test and 0% after genetic confirmation was shown. The prevalence of CAH in this cohort of adrenal incidentalomas was thus similar to that in the general population of 0.1% (23), which contrasts our hypothesis that the prevalence should be higher than that in the general population. However, one of the eight subjects presented a pathological *CYP21A2* variation in one of the two alleles. The prevalence of being a *CYP21A2*-variation carrier was therefore 0.5% (1 of 222) in our study population.

A meta-analysis based on 36 publications reported that 58 of 990 (5.9%) subjects with AI were diagnosed biochemically with CAH. The prevalence of CAH based on gene analysis was only 0.8% (2 of 252). The frequency of carriers were 10.2% (36 of 352) (26). Our data, with a prevalence of 0% of CAH and 0.5% of carriers, do not match the findings. Therefore, compared with previous studies in this field, our data do not clearly speak for a higher prevalence of NC CAH in a population with AI (26). An important factor that likely had a large effect on the outcome in our study is the composition of different ethnicities in this cohort. The prevalence of NC CAH is probably lower in a cohort with Swedish ethnicity than in many other populations. Other studies with higher prevalence of NC CAH were probably based on more multi-ethnic populations (14, 27). Having said that, the p.Val281Leu variant, which was found in the individual being a carrier in the current study, is the most common gene variations in NC CAH (23).

This population in regional Sweden, on which the study cohort was extracted from, likely had a lower proportion of multi-ethnicity compared with metropolitan regions in Sweden. As an example, in 2021, 20% of Sweden’s total population was foreign-born compared to 26.5% in Stockholm and 13% in the region where our study was performed (28). The even higher frequency of Swedish ethnicity, 94.1% in the study cohort, is likely explained by the fact that the proportion of foreign-born or with foreign background was lower at older ages, especially in regional Sweden, where the majority of adrenal incidentalomas were diagnosed. This relationship may be the most important explanation to the failure to detect any case of genetically confirmed CAH in our study.

As no case of CAH could be confirmed among the eight subjects with stimulated serum 17OHP levels ≥30 nmol/L after analysis of the *CYP21A2* gene, our data underline the importance of confirming the CAH diagnosis with gene analysis, especially in mildly elevated 17OHP levels after ACTH stimulation in patients with adrenal tumors. This is in accordance with the previously mentioned meta-analysis (26).

Another aim of the study was to investigate if there were any clinical, radiological, or biochemical characteristics of individuals with CAH differentiating them from individuals without CAH. As no cases of CAH could be detected in our cohort, we were unfortunately unable to achieve this aim. However, it seems more likely to detect elevated levels of stimulated serum 17OHP in individuals with bilateral compared with unilateral lesions. The frequency of bilateral lesions was higher in cases with serum 17OHP levels ≥30 nmol/L than in cases with lower 17OHP levels, 50% vs. 10.3%, or in other words, the probability to detect a stimulated serum 17OHP level ≥30 nmol/L in bilateral lesions was 0.15 (4 of 26) compared with 0.02 (4 of 196) in unilateral lesions. These findings support the ESE/ENSAT recommendation to consider CAH in bilateral lesions (8), although no case of CAH could be ascertained among our individuals with a serum 17OHP level ≥30 nmol/L. Moreover, we found a correlation between 17OHP levels and tumor size further complicating the situation and stressing the importance of confirming the NC CAH diagnosis in individuals with mildly elevated 17OHP levels and adrenal tumor(s).

Four cases with 17OHP levels ≥30 nmol/L had unilateral lesions, and no pathological *CYP21A2* variation was detected in any of the subjects with unilateral lesions. However, even though we did not find any case of genetically verified CAH or carrier with unilateral lesions, they do exist, and CAH could not be excluded only by the fact that it is a unilateral lesion (24, 26). A possible explanation of the elevated 17OHP levels in these subjects is the intratumoral defects of steroidogenesis, i.e., an acquired defect of 21-hydroxylase in tumor cells alone without detectable pathological *CYP21A2* variations on blood analysis. However, to confirm this theory, it would be necessary to demonstrate the presence of dysfunctional 21-hydroxylase (i.e., *CYP21A2* variations) in the tumors, which would require adrenalectomy.

Among the typical clinical symptoms and findings in CAH, neither the subject with a detected *CYP21A2* variation nor the other seven subjects with serum 17OHP levels ≥30 nmol/L presented hyperandrogenic signs or symptoms as expected, since none of them were classified as CAH after *CYP21A2*-gene analysis. Furthermore, none of the eight subjects classified as CAH after ACTH stimulation presented a history of cortisol deficiency symptoms and demonstrated, as expected, also sufficient cortisol levels basically and after ACTH stimulation. These findings are in line with known facts about CAH, since only the two more severe forms, SW and SV CAH, usually present with hyperandrogenism and adrenal insufficiency in contrast to NC CAH (19–21). The subject with a detected *CYP21A2* variation had a hyper-responsivity to cortisol after ACTH stimulation and a lack of suppression to DST. The hyper-responsivity to cortisol after ACTH stimulation has previously been shown in patients with a heterozygotic variation in *CYP21A* gene and is thought to be caused by upregulated ACTH activity due to the relative deficiency in cortisol production and feedback inhibition. This prompt cortisol response has been hypothesized to be favorable, since it may also enable a more rapid return to homeostasis and is thought to be the reason for the lower mortality from severe infections found in patients with a detected *CYP21A2* variation (29). The lack of suppression in the patient with a detected *CYP21A2* variation in the current study may be due to the patient being a quick metabolizer of dexamethasone causing a decreased suppression of cortisol, and possibly another contributing factor could be the tumor size, since there is a correlation between tumor size and post-DST cortisol levels.

Regarding other potential biomarkers for CAH, there was a significant difference in ACTH-stimulated serum cortisol levels between subjects with 17OHP levels ≥30 nmol/L and those with lower 17OHP levels. The stimulated cortisol levels were significantly higher in the group with 17OHP levels ≥30 nmol/L (p =0.030), which is probably explained by the correlation between tumor size and stimulated cortisol levels, when the tumor size was significantly larger in the group with higher levels of 17OHP. As we did not identify any individuals with CAH, these findings are in accordance with established knowledge that the level of cortisol after ACTH stimulation gives no additional support in screening for CAH in patients with AI (21, 30). However, upon detection of CAH, the stimulated cortisol level is an indicator for the need of continuous glucocorticoid replacement (15, 20, 21, 23, 29).

DHEAS is an androgenic precursor that may be elevated in classic CAH (31), but serum DHEAS levels are not considered as a primary biomarker in CAH screening (15, 19–21, 30, 32, 33). None of the eight subjects with a 17OHP level ≥30 nmol/L presented an elevated DHEAS level. An elevated DHEAS level is, although primary, expected to be observed in un- or undertreated classic CAH (21, 33, 34). No case of classical CAH could be identified in our study, which explains that none of the eight cases undergoing gene analysis presented an elevated serum DHEAS level. Thus, our data are in line with previous studies and do not support the use of an elevated serum DHEAS level as a biomarker for undiagnosed CAH in the clinical management of AIs.

There are other potential biomarkers than the traditional serum 17OHP levels that may be used for screening of CAH in the clinical management of AIs. The steroids 11-deoxycortisol, 11-ketotestosterone, 21-dexycortisol, and 11-hydroxytestosterone may be more sensitive biomarkers than the traditional serum 17OHP levels, with the potential to facilitate diagnostics of NC CAH (31, 35–38).

Furthermore, an ACTH-stimulated 17OHP level is usually necessary to diagnose NC CAH (21, 23), which requires staff resources and extended time for the patient compared to basal blood tests. Thus, these new biomarkers for CAH may have the potential to be more accurate than 17OHP and simplify the screening for CAH. However, to date, no work has been published using any of these biomarkers to screen for CAH in a cohort of AIs. Moreover, the availability to analyze these steroids is limited in most clinical laboratories.

The strength of our study is the prospective design with screening of, in contrast to previous studies, a large population fulfilling the established criteria of AI in combination with the use of a well-established screening method for CAH, the ACTH-stimulation test. Gene analysis to detect pathological *CYP21A2* variations in cases classified as CAH after ACTH stimulation provides further strength. The study was performed at a regional hospital with a study population more representative of the normal population and not explicitly selected that may be a risk at a university hospital. The main weakness of our study is the fact that gene analysis was not performed on the entire study population. A complete analysis of the *CYP21A2* gene is a relatively expensive and cumbersome analysis. However, CAHs are diagnosed primarily with 17OHP levels, and patients with CAH due to 21OHD will always have a 17OHP above 30 nmol/L, so we are quite sure that we have not missed any case of CAH due to 21OHD. In contrast, 17OHP levels may be falsely elevated due to the adrenal tumor *per se*, i.e., the prevalence of CAH could be overestimated if no genetic analysis was done. Furthermore, the size of the study population after the completion of the study may be perceived as too small, as we could not identify any cases with *CYP21A2* variations on both alleles. We do not think that the size of the study population was miscalculated, as previous studies in the field have shown clearly a higher prevalence of both CAH and *CYP21A2*-variation carriership despite significantly smaller study populations compared with ours (26). Another known weakness mentioned above was that ACTH-stimulated 17OHP levels may be above cutoff (30 nmol/L) even without detectable *CYP21A2* variations. There are previous data supporting that serum 17OHP levels after ACTH stimulation can be elevated in adrenal adenomas without the presence of 21OHD (39–43) and that the rise is correlated to tumor size. However, this potential issue was excluded by carrying out gene analysis on all subjects with elevated 17OHP levels.

Theoretically, there is also a risk of missing CAH in individuals with other enzyme defects than 21OHD when only 17OHP levels were measured and gene analysis were performed of the *CYP21A2* gene only. It should be noted that 11β-hydroxylase deficiency, a rare CAH variant, can present with increased 17OHP levels and then be misdiagnosed as 21OHD (44). However, other enzyme defects than 21OHD are extremely rare in a Caucasian population (16, 17), and none of the subjects with an elevated 17OHP level were of non-Caucasian ethnic origin. Therefore, we assume that analysis of only 17OHP levels and then the *CYP21A2* gene was not an issue that could affect the results of our study. Thus, the risk of overestimating the prevalence of CAH with this study design is negligible. However, an underestimation of the true prevalence is possible with our study design, mainly due to the population size and the fact that gene analysis was not performed in all subjects.

To summarize, CAH seems to be a very rare cause of AI in a mostly Swedish Caucasian population. Screening with stimulated 17OHP levels does not appear warranted and may cause overdiagnosis of NC CAH if no genetic analysis is done. However, this does not mean that CAH should not be considered in a patient with AI, especially in an ethnicity known to have increased prevalence of NC CAH. The diagnosis should be considered in every patient, but screening for CAH should only be performed if it is beneficial for the individual patient or of importance for the clinical management, e.g., in premenopausal women. There is still a need to find easier ways for clinicians to identify individuals who should be investigated for CAH and more accurate and easier methods to biochemically diagnose CAH than basal and ACTH-stimulated serum 17OHP levels.

## Data availability statement

The original contributions presented in the study are included in the article/supplementary material. Further inquiries can be directed to the corresponding author.

## Ethics statement

The studies involving human participants were reviewed and approved by The Regional Ethical Review Board in Uppsala, Sweden [diary number 2015/096 (2019-04522)]. The patients/participants provided their written informed consent to participate in this study. Written informed consent was obtained from the individual(s) for the publication of any potentially identifiable images or data included in this article.

## Author contributions

FS wrote the manuscript, collected data, and performed the statistical analysis. HF and SB analyzed data, contributed to the discussion, and reviewed and edited the manuscript. All authors critically reviewed the manuscript. FS had full access to all the data in this study and takes responsibility for the integrity of the data in the study and the accuracy of the data analysis. All authors contributed to the article and approved the submitted version.

## Funding

This study was financed by grants from the Center for Clinical Research Dalarna-Uppsala University (grant numbers CKFUU 796881, 898811, 934155, 936642 and 963295) and Magnus Bergvall Foundation (grant numbers 2018-02566, 2019-03149, 2020-03724, and 2021-04226).

## Acknowledgments

We would like to thank the Center for Clinical Research Dalarna-Uppsala University for their financial and logistic support. Massive thanks to our colleagues in Region Dalarna, especially at the Department of Radiology, for their interest in and contribution to our study by providing potential subjects for the study. Also thanks to the Department of Laboratory Medicine Clinical Chemistry for their support. Finally, above all, a special thanks to the staff at the Department for Diabetes and Endocrinology at Falu Hospital for their interest, patience, and logistical support. Without their support, this study would not have been feasible.

## Conflict of interest

The authors declare that the research was conducted in the absence of any commercial or financial relationships that could be constructed as a potential conflict of interest.

## Publisher’s note

All claims expressed in this article are solely those of the authors and do not necessarily represent those of their affiliated organizations, or those of the publisher, the editors and the reviewers. Any product that may be evaluated in this article, or claim that may be made by its manufacturer, is not guaranteed or endorsed by the publisher.

## Abbreviations

ACA, adrenocortical adenoma; ACC, adrenocortical carcinoma; ACTH, adrenocorticotrophic hormone; ACS, autonomous cortisol secretion; AI, adrenal incidentaloma; CAH, congenital adrenal hyperplasia; CT, computer tomography; ESE, European Society of Endocrinology; HU, Hounsfield units; MRI, magnetic resonance imaging; NC, non-classic; NC CAH, non-classic congenital adrenal hyperplasia; PACS, possible autonomous cortisol secretion; SV, simple virilizing; SW, salt wasting; 17OHP, 17-hydroxy progesterone; 21OHD, 21-hydroxylase deficiency.
